# Estimation of daily aluminum intake in Japan based on food consumption inspection results: impact of food additives

**DOI:** 10.1002/fsn3.114

**Published:** 2014-04-20

**Authors:** Kyoko Sato, Ippei Suzuki, Hiroki Kubota, Noriko Furusho, Tomoyuki Inoue, Yoshikazu Yasukouchi, Hiroshi Akiyama

**Affiliations:** 1 Division of Food Additives, National Institute of Health Sciences1-18-1 Kamiyoga, Setagaya-ku, Tokyo, 158-8501, Japan; 2 Japan Frozen Foods Inspection Kansai Office3-2-6 Minatojima, Minamimachi, Chuo-ku, Kobe, Hyogo, 650-0047, Japan

**Keywords:** Aluminum, dietary intake, food additive, ICP-AES, provisional tolerable weekly intake

## Abstract

Dietary aluminum (Al) intake by young children, children, youths, and adults in Japan was estimated using the market basket method. The Al content of food category (I–VII) samples for each age group was determined by inductively coupled plasma-atomic emission spectrometry (ICP-AES). The Al content in processed foods and unprocessed foods ranged from 0.40 to 21.7 mg/kg and from 0.32 to 0.54 mg/kg, respectively. For processed foods in all age groups, the Al content in food category VI samples, sugar and confections/savories, was the highest, followed by those in category II, cereals. The daily dietary Al intake from processed foods was much larger than that from unprocessed foods. The mean weekly percentages of the provisional tolerable weekly intake (PTWI, established by the joint FAO/WHO Expert Committee on Food Additives in 2011) from processed foods for all age groups are 43.1, 22.4, 17.6 and 15.1%, respectively. Only the highest consumer Al exposure value (>*P*_95_) of the young children group exceeded the PTWI.

## Introduction

Aluminum (Al) is the third most abundant element in the earth's crust and is widely applied to human life. However, the toxic effects of Al have been reported in several studies. Many researchers have reported that Al can be toxic to the central nervous, skeletal, and hematopoietic systems, and Al has been controversially implicated in Alzheimer's disease, osteomalacia, and dialysis encephalopathy (Nordberg et al. 2007; Aguilar et al. 2008; Peto 2010; She et al. 2012), although this remains to be clearly demonstrated.

The joint FAO/WHO Expert Committee on Food Additives (JECFA) established the provisional tolerable weekly intake (PTWI) for Al of 7.0 mg per kg body weight in 1989 (Joint FAO/WHO Expert Committee on Food Additives 1989). In 2007, the JECFA re-evaluated the safety of Al and lowered the PTWI to 1.0 mg per kg body weight because of the potential for Al to affect the reproductive and nervous system in experimental animals (Joint FAO/WHO Expert Committee on Food Additives 2007). However, the JECFA revised the PTWI to 2.0 mg per kg body weight in 2011 (Joint FAO/WHO Expert Committee on Food Additives 2011) as a result of new bioavailability and toxicological data (Poirier et al. 2011).

Al in the food supply comes from natural sources, the water used in food preparation, food ingredients, and utensils used during food preparation. The primary dietary sources of Al are foods and beverages, and these sources are either naturally existing or intentionally added (Pennington 1988; Greger 2007; Aguilar et al. 2008). Many researchers have reported estimates of Al dietary exposure in countries and regions, such as the United States (Pennington and Schoen 1995), Greece (Bratakos et al. 2012), Belgium (Fekete et al. 2013), South China (Jiang et al. 2013) and the European Union (Aguilar et al. 2008). However, there are large variations in Al exposure among the different countries and regions. These variations are due to differences in the survey methodologies employed, which take into account differences in population, dietary patterns, and consumption, as well as the reported Al concentrations in foods prepared using Al-containing food additives. Matsuda et al. (2001) reported that the average daily dietary Al intake in Japan, estimated from a total diet study between 1996 and 1998, was 3.5 mg, with a range of 1.8–8.4 mg. However, this estimated dietary Al intake is based on data collected more than 10 years ago and does not reflect current dietary intake. Therefore, it is necessary to assess the current dietary Al exposure in Japan in order to regulate Al intake from Al-containing food additives, given the JECFA revisions to the current PTWI exposure guidelines for Al.

In the present study, we determined the Al content of various food categories and its relation to Al intake by different age groups in Japan (young children, children, youths, and adults) using the market basket method. Samples were analyzed using inductively coupled plasma-atomic emission spectrometry (ICP-AES). The weekly dietary Al exposure from processed and unprocessed foods for all age groups was then estimated. Furthermore, the highest levels of consumer weekly Al exposure from processed foods for all age groups were estimated. Additionally, we discuss the estimated values and compare them with the JECFA revision to the PTWI in 2011.

## Material and Methods

### Reagents and chemicals

Hydrochloric acid (HCl) (35.0–37.0%) for measurement of toxic metals, nitric acid (HNO_3_) (60–62%) for precision analysis, 0.1 mol/L HNO_3_ for quantitative analysis, Al standard solution (100 *μ*g/mL, certified by Japan Calibration Service System), and Ultrapure Water (H_2_O) were purchased from Wako Pure Chemical Industries (Osaka, Japan). Calibration standard solutions of Al (0.012, 0.06, 0.3, 1.5, and 7.5 *μ*g/mL) were prepared by dilution of Al standard solution with 0.1 mol/L HNO_3_.

### Food consumption data and market basket method

The daily dietary Al intake was estimated using the market basket method (Ishiwata et al. 2002). For processed foods, a daily food consumption list of 189 types of processed foods in seven food categories for the four age groups was prepared using the data on the usual daily diet in Japan from the Special Survey Study of the Frequency and Level of Food Consumption (2005–2007) (government closed report), which was based on individual food intake surveys. The Special Survey Study calculated the average daily consumption of 189 types of food by the following age groups: 1–6 year olds (young children), 7–14 (children), 15–19 (youths), and older than 20 years (adults). The 189 food types are classified into seven food categories (I, seasonings and beverages; II, cereals; III, potatoes, legumes, nuts; IV, fish/shellfish, meat and hens' eggs; V, oils/fats and milk/milk products; VI, sugar and confections/savories; VII, fruits, vegetables, and seaweeds). Based on the prepared list, 286 processed food products (189 types of processed food) were purchased from a supermarket in Tokyo. For popularly consumed processed foods, several different brands were purchased, allowing the actual conditions of consumption (frequency and level of consumption) and processing methods to be taken into consideration.

We measured the required amounts of individual food products consumed by each age group, according to the Special Survey Study list. The collected food samples for each category were mixed, yielding 28 samples (4 [age groups] × 7[food categories]). Briefly, for each food category (I–VII), a predetermined amount of food was weighed for each food category for each age group. The amount for a specific age group for that particular food category was then homogenized with an equal weight of ultrapure water to yield a single sample using a blender (Knife Mill GRINDOMIX GM 300; Retsch, Haan, Germany). The one exception was food category I, which was homogenized without added water. In the Results and Discussion section, the determined Al concentration in each food category is shown as the amount of Al for the weight of the sample without added water. The 28 homogenized samples were separately bottled in 100 mL antioxidant-free polyethylene bottles and frozen below −20°C until analyzed.

For the unprocessed foods, we prepared a daily food consumption list of 33 types of unprocessed foods from four food categories (II, III, IV, and VII) based on the food consumption in the All Investigation Report of Daily Intake of Food Additives in 2000 (http://www.ffcr.or.jp/Zaidan/mhwinfo.nsf/98a5d7b766af9bfb492565a10020c601/1100115ac3d23fd7492569d800171034?OpenDocument). Forty unprocessed food products (33 types of unprocessed foods) based on the All Investigation Report were purchased from a supermarket in Kanagawa Prefecture or through the internet.

We measured the required amounts of individual food products for each age group, according to the list for unprocessed foods. The measured food samples for each category were mixed, yielding 13 samples (4 [age groups] × 3 [food categories]) and a sample of food category III (since the sample for food category III is identical for all age groups). Briefly, for each food category (II, IV, and VII), a predetermined amount of food was weighed for each age group; then, all the food was homogenized to yield a single sample for each food category for each age group using blenders (Ultra Centrifugal Mill ZM 200, Knife Mill GRINDOMIX GM 300; Retsch) and BLIXER-5Plus (FMI Co., Osaka, Japan) and a food processor (MK-K58; Panasonic Co., Kadoma, Japan). The homogenized samples were separately bottled in 125.0 mL antioxidant-free polyethylene bottles and frozen below −20°C until further analyses.

### Preparation of test solutions

Aliquots (2.0 g) of the homogenized samples were weighed and transferred into quartz beakers. The samples were dried and carbonized on a hotplate at 250°C. The carbide was then placed in a furnace and the temperature was raised to 485°C at 100°C/h and kept at 485°C overnight. After cooling, 5.0 mL of HNO_3_/H_2_O (1:1) mixture was added and the mixture was dried on the hotplate and incinerated in a furnace. The ash was wetted with H_2_O, and 5.0 mL HCl was added and dried on the hotplate. The ash was dissolved with 0.1 mol/L HNO_3_ and the volume was adjusted to 50.0 mL using Digi TUBEs (SCP SCIENCE Co., Baie-D'Urfé, Canada).

### Al determination

Al concentrations in the food categories were analyzed using an Optima 5300DV ICP-AES (Perkin-Elmer, Waltham, MA). The ICP-AES operating condition parameters were: liquid uptake, 1.0 mL/min; plasma argon gas flow, 15.0 L/min; auxiliary argon gas flow, 0.2 mL/min; carrier gas flow, 0.7 mL/min; RF generator, 1300 W; optical viewing axially observed spectral lines, 395.153 nm.

### Determination of dietary Al intake from processed and unprocessed foods

The daily Al intake, based on the Al concentration determined using ICP-AES for the food category samples and the daily consumption of each food category by each age group, was calculated using the following equations.


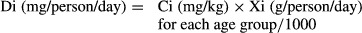



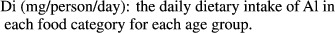



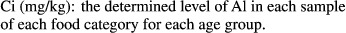



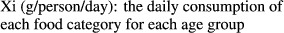


The daily intake of the processed food (categories I–VII) and unprocessed foods (categories II, III, IV, and VII) was separately determined and summed as the subtotal daily intake for that food category.

Total daily intake of Al for each age group was calculated using the following equation.


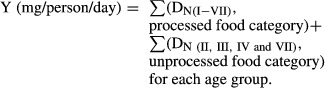


The weekly Al intake for each age group was calculated using the following equation:


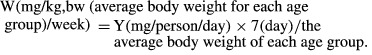


The mean body weight of each age group is 16.0 kg for young children, 36.5 kg for children, 56.5 kg for youths, and 58.6 kg for adults.

### Percentile analysis of Al exposure

Percentile analysis of Al exposure from processed foods for each age group was conducted to assess high-level dietary Al exposure in consumers. The *P*_50,_ *P*_95,_ *P*_97.5,_ and *P*_99_ dietary Al exposures were calculated as follows. The 287 types of processed foods in the individual daily food consumption data of the Special Survey Study of the Frequency and Level of Food Consumption (2005–2007) [government closed report], based on data from four age groups (young children [1–6 years old]: *n* = 1619; children [7–14 years old]: *n* = 3419; youths [15–19 years old]: *n* = 2539; adults [older than 20 years old]: *n* = 32787), were categorized into food categories I–VII. The individual consumption of each food category was calculated. The individual Al intake from the processed foods was then calculated by multiplying the individual consumption of each food category for I to VII food categories and the determined level of Al in that food category. Next, to calculate the individual daily Al intake (mg/kg bw/day), the individual Al intake from the processed food categories (mg/day) was divided by the individual body weight. The individual weekly Al intake (mg/kg bw/day) was calculated by multiplying by 7 (days). The percentile value of the individual weekly Al intake (*P*_50,_ *P*_95,_ *P*_97.5_, and *P*_99_) was extracted from the data of the individual weekly Al intake.

## Results and Discussion

### Validation method

The limit of detection (LOD) and limit of quantification (LOQ) of the developed method for Al determination was estimated based on the standard deviation (*σ*) of seven independent measurements of food category IV samples for processed foods (children) and food category II samples for unprocessed foods (young children), according to the quality assurance guideline announced by the Japanese Ministry of the Environment (2003). The LOD and LOQ are estimated to be 0.1 mg/kg (<3.143 *σ*) and 0.3 mg/kg (<9.429 *σ*), respectively.

The accuracy and precision of the developed method for Al determination were evaluated by recovery tests using three independent measurements of all food category samples for the adult age group for processed foods, and food category II, III, IV, and VII samples for all age groups for unprocessed foods.

For the mixed processed food samples of all food categories, the recovery of Al ranged from 83% to 113%, with a relative standard deviation (RSD) of <7.8%. The results of the recovery tests are shown in Table 1. For the mixed unprocessed food samples for categories II, III, IV, and VII, Al recovery with the level of quantification limit (0.3 mg/kg as Al) ranged from 81% to 106%, with an RSD of <18.5% (data not shown). The results show that the developed method is acceptable for determining the total Al concentration in all food samples in this study.

**Table 1:** Results of spike and recovery tests for each food group

Food group	Spiked aluminum amount (mg/kg)	Recovered (%)	Relative standard deviation (%)
I	2	113.2	1.8
II	3	83.0	1.6
III	0.5	101.8	2.9
IV	0.5	106.9	6.6
V	0.5	84.5	7.8
VI	10	101.1	3.5
VII	3	92.8	3.7

### Determination of Al concentration in processed and unprocessed foods using the market basket method

To estimate the Al content in processed foods, we determined the Al content of all food category samples for all age groups. As shown in Table 2, the Al content of processed food samples in all food categories ranged from 0.40 to 21.7 mg/kg. For samples of processed food categories for all age groups, Al content followed the rank order: category VI samples > II > VII > III. The main source of Al in category VI foods is thought to be food additives, such as leavening agents. In the category VI processed foods examined (55 products), 22 products were labeled as containing baking powder. In the category II processed foods examined (40 products), four products were labeled as containing a leavening agent. The Al detected in category VII foods is thought to be derived from natural sources such as soil and seawater. Researchers have reported increasing environmental contamination to be the cause of vegetable- and fruit-derived Al (Müller et al. 1998; Jiang et al. 2013), and that Al is derived from food additives such as aluminum ammonium sulfate and aluminum potassium sulfate used as color-fixing agents.

**Table 2:** Al concentrations in processed foods (mg/kg)

	Food group						
	
	I	II	III	IV	V	VI	VII
							
Age group	Seasonings and beverages	Cereals	Potatoes, legumes, nuts	Fish/shellfish, meat and eggs	Oils/fats and milk/milk products	Sugar and confections/ savories	Fruits, vegetables and seaweeds
Young children	0.67	6.57	1.13	0.46	1.21	21.73	1.56
Children	0.76	5.49	1.07	0.41	1.09	17.03	1.68
Youths	0.86	7.68	1.05	0.66	1.24	20.57	2.35
Adults	0.99	4.93	1.13	0.40	0.49	19.12	4.45

The Al detected in the category III foods is also thought to be derived from soil and other environmental factors because none of the labels on the category III foods indicated food additives containing Al. Furthermore, legumes such as lentils and red beans, raisins, and nuts such as almonds and hazelnuts, are reported to be rich sources of Al (Wang et al. 1991; Soliman and Zikovsky 1999; Cabrera et al. 2003; Bratakos et al. 2012).

The Al content in category IV foods containing processed fish products was lower than that of category III foods. In a previous Japanese study, Al levels (3.70–10.9 mg/kg) in fish samples were rather high (Matsuda et al. 2001). The high Al levels in processed fish products previously reported might be due to the presence of added stabilizers, such as food additives like aluminum ammonium sulfate and aluminum potassium sulfate, used in processed fish products in Japan, or due to differences in the varieties of fish used to manufacture the processed fish products.

To estimate the Al content from unprocessed foods, we further determined the Al content of unprocessed food samples of food category II, III, IV, and VII samples for all age groups. As shown in Table 3, the Al content of samples of unprocessed foods in these categories ranged from 0.32 to 0.54 mg/kg. The results show that Al is widely contained in processed and unprocessed foods. Furthermore, the Al content in processed foods appears to be much higher than that in unprocessed foods. These results are consistent with previous reports (Jiang et al. 2013).

**Table 3:** Al concentrations in unprocessed foods (mg/kg)

	Food group			
	
	II	III	IV	VII
				
Age group	Cereals	Potatoes, legumes, nuts	Fish/shellfish, meat and eggs	Fruits,vegetables and seaweeds
Young children	0.32	0.43	0.40	0.37
Children	0.43	0.43	0.33	0.50
Youths	0.36	0.43	0.44	0.50
Adults	0.39	0.43	0.33	0.54

For unprocessed category II foods (cereals), the main cereal consumed by Japanese is rice. The expected Al levels in rice are consistent with the present results (0.32–0.43 mg/kg), but are considerably lower than the category III levels reported in Germany (8.5 mg/kg) (Müller et al. 1998), which were obtained for potatoes. However, the current result is similar to the levels (0.64 mg/kg) in potato reported in Greece (Bratakos et al. 2012). It should be noted that the differences between the values obtained in the present study and the reported values could be due to the environmental conditions in which the crops were grown.

In this study, the Al levels of food category IV samples, including unprocessed fish/shellfish, meat and hens' eggs, were less than 0.50 mg/kg. These results are similar to those previously reported (Bratakos et al. 2012; Fekete et al. 2013). It was reported that the Al levels in fish samples were below 1.0 mg/kg, whereas those in fresh meat were below 0.40 mg/kg. The Al levels in hens' eggs and egg products were reported to be less than 0.74 mg/kg (Pennington 1988; Müller et al. 1998; Soliman and Zikovsky 1999; Bratakos et al. 2012; Jiang et al. 2013).

For unprocessed category VII foods (fruits, vegetables, and seaweeds), the highest Al levels were present in fruits and vegetables. The present levels (0.37–0.54 mg/kg) were similar to the levels (typically less than 0.80 mg/kg) reported in Greece (Bratakos et al. 2012), and lower than the levels (2.41 mg/kg for fruits, 1.96 mg/kg for vegetables) reported in Belgium (Fekete et al. 2013). However, the Al levels in fruits and vegetables previously reported varied significantly (0.07–41.1 mg/kg) (Müller et al. 1998; Bratakos et al. 2012; Fekete et al. 2013; Jiang et al. 2013).

### Estimation of dietary Al intake

Dietary Al exposure is related to both Al levels in foods and amount of food consumption. Therefore, the estimate of daily dietary Al intake was based on both the results of Al levels in food categories estimated by the market basket method, and the listed dietary intake amounts of various foods per age group. The daily dietary Al intake of processed and unprocessed foods is shown in Tables 4 and 5, respectively. As shown in Table 4, adults have the highest Al intake from food category I, youths and children showed the highest intake from food category II, while young children showed the highest intake from food category VI. For unprocessed foods, the dietary Al intake from food category VII was the highest for all age groups (Table 5).

**Table 4:** Daily Al intake from processed foods (mg/person/day)

	Food group						
	
	I	II	III	IV	V	VI	VII
							
Age group	Seasonings and beverages	Cereals	Potatoes, legumes, nuts	Fish/shellfish, meat, and eggs	Oils/fats and milk/milk products	Sugar and confections/ savories	Fruits, vegetables, and seaweeds
Young children	0.23	0.56	0.08	0.01	0.09	0.83	0.01
Children	0.35	0.74	0.11	0.02	0.10	0.70	0.02
Youths	0.49	1.00	0.10	0.03	0.10	0.75	0.03
Adults	0.70	0.59	0.14	0.02	0.03	0.57	0.11

**Table 5:** Daily Al intake from unprocessed foods (mg/person/day)

	Food group			
	
	II	III	IV	VII
				
Age group	Cereals	Potatoes, legumes, nuts	Fish/shellfish, meat and eggs	Fruits, vegetables, and seaweeds
Young children	0.03	0.02	0.04	0.08
Children	0.05	0.03	0.06	0.17
Youths	0.07	0.02	0.09	0.17
Adults	0.07	0.02	0.06	0.22

**Figure 1:**
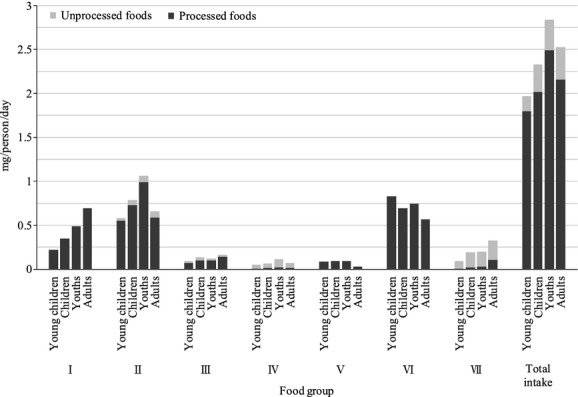
Total daily Al intake. The graph indicates the estimated daily intake of Al from each food category. Al intake from processed foods is indicated in black, and Al intake from unprocessed foods is indicated in gray.

Total daily Al intake from processed and unprocessed foods is shown in Figure 1. The daily dietary Al intake from processed foods is much larger than from unprocessed foods, and total daily Al intake in youths was the highest among all age groups. The study indicated that processed foods from food categories I, II, and VI significantly contribute to Al intake; however, the ratio of daily intake from unprocessed foods to the subtotal daily intake for food categories IV and VII is larger (77–79% for category IV foods and 67–88% for category VII foods). These findings suggest that the daily Al intake can be roughly estimated using only the processed food data for each age group.

The results of dietary Al intake estimates obtained in this study were similar to recent results from other national investigations, such as in Greece, China, and Belgium, although the survey measures of each investigation are unique and different. It was reported that nonalcoholic beverages are the most important contributor to Al intake in the Belgian adult population (Fekete et al. 2013). The present results show that the greatest contributor to Al intake in the adult Japanese group also appears to be beverages: the food group encompassing nonalcoholic beverages corresponds to food category I in this study (Fig. 1). In Greece, researchers reported that the major Al contributors are cereal products and vegetables, corresponding to 46.7% and 25.7% of total Al intake, respectively (Bratakos et al. 2012). The present results show that the major contributor to Al intake in the total group also appears to be cereal products, which correspond to food category II in this study. We also showed that category VI foods, such as sugar and confections/savories, in this study are major contributors to Al intake in the total group. However, confectionery products are small contributors (5.3%) to Al intake in Greece (Bratakos et al. 2012). We classified food products such as cake doughnut, butter cake and steamed bun as confectioneries, and placed them in food category VI. Food products similar to cake doughnut and butter cake, but not steamed bun, are consumed in Greece. These food products might be classified in cereal products in the study in Greece. Therefore, the observed differences for sugars and sweets between the study of Greece and the present study might be due to differences in food group categorization, and/or differences in consumption, and/or differences in food additive levels. The unique food products such as steamed bun are consumed mostly in Asian counties. Moreover, a similar study in South China showed that steamed flour products typically contain quantities of Al and are a major contributor to Al intake (Jiang et al. 2013). The Chinese study suggests that steamed flour products in South China contain food additives, including Al as leavening agents. The result of our study is thought to be consistent with a study in South China. While the daily Al intake in this study is significantly lower than that indicated in a previous Japanese report from 2001, the food categories that are the main contributors to daily Al intake in this study are quite similar to those of a previous report (Matsuda et al. 2001).

### Determination of Al levels in individual food products of the high Al content food categories

To investigate in detail the kinds of processed food products contributing to high Al consumption, we determined the Al levels of individual processed food products in food categories I, II, and VI that are the main contributors to Al intake. The levels of Al and the daily dietary Al intake for all age groups are shown in Tables S1–S3. In food category I (Table S1), the processed food with the highest Al level was curry roux (10.7 mg/kg), followed by milk cocoa (9.69 mg/kg), and soluble coffee powder (6.12 mg/kg). In food category II (Table S2), the processed food with the highest Al level was tempura flour (222 mg/kg), followed by sweetened bun (156 mg/kg), Chinese steamed bun with a bean-jam filling (59.2 mg/kg), and Chinese steamed bun with meat filling (57.5 mg/kg). In food category VI (Table S3), the processed food with the highest Al level was cake doughnut (258 mg/kg), followed by Japanese steamed bun (172 mg/kg) and butter cake (116 mg/kg). The range of Al concentrations in the various food products examined was 0.95–172 mg/kg for Japanese steamed bun products, 1.53–258 mg/kg for cake doughnut products, less than 0.3–59.2 mg/kg for shortcake products, and less than 0.3–116 mg/kg for butter cake products. In the present study, Chinese steamed bun is classified as a category II food because the product is processed using baker's yeast, and Japanese steamed bun is classified as a category IV food because the product is produced without using baker's yeast. Al levels in the present study are in line with previously reported results (Matsuda et al. 2001; Fekete et al. 2013).

In regions and countries such as the EU and the USA, sodium aluminum phosphate, used as a leavening agent in cereals and related products, and/or sodium aluminum silicate used as an anticaking agent in cake mixes and dried products, contribute highly to Al levels in wheat bread, cakes, cookies, and confectionaries. Food additives such as sodium aluminum phosphate and sodium aluminum silicate are approved for use as food additives (Jorhem and Haegglund 1992; Yang et al. 1994; Leblanc et al. 2005; Saiyed and Yokel 2005; Bratakos et al. 2012) in the USA and EU countries. However, in Japan, sodium aluminum phosphate and sodium aluminum silicate are not currently approved for use as food additives, whereas aluminum ammonium sulfate and aluminum potassium sulfate are currently used as food additives, without limits on the amounts used. Therefore, in Japan, the high Al levels in some samples of both categories II and VI might be attributed to the use of food additives such as aluminum ammonium sulfate and aluminum potassium sulfate as leavening agents.

In food category I, major contributors to daily Al intake include vegetable juice for young children (0.02 mg/person/day), curry roux for children (0.04 mg/person/day), and green tea for youths and adults (0.07 mg/person/day for youths and 0.16 mg/person/day for adults). For food categories II and VI, sweetened bun and cake doughnut are the main contributors to the intake of Al for all age groups. We suggest that the high Al levels in these processed foods contribute to high Al intake.

### Percentile analyses of Al exposure in each age group

The weekly dietary Al intake due to processed foods was calculated by multiplying the daily Al intake value by 7 (7 days/week). The average weekly dietary Al exposure from processed foods for young children, children, youths, and adults is estimated to be 0.86, 0.45, 0.35 and 0.30 mg/kg bw/week, respectively. The percentages of those for all age groups to the PTWI established by the JECFA in 2011 are 43.1, 22.4, 17.6 and 15.1%, respectively.

**Figure 2:**
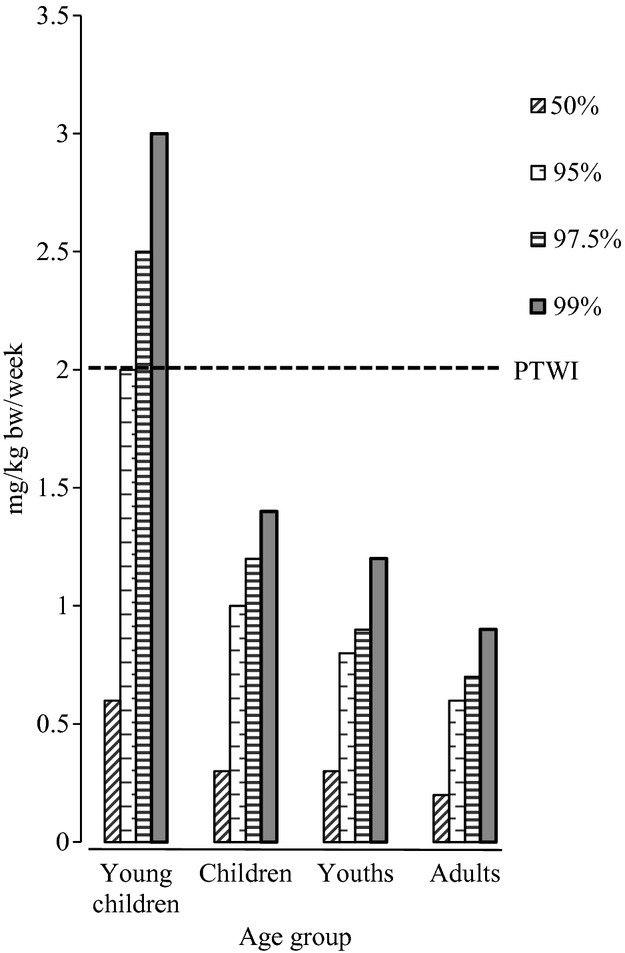
Percentile analysis of Al intake from foods. Each column indicates the estimated weekly Al intake for each percentile value. The dashed line indicates provisional tolerable weekly intake (PTWI; 2 mg/kg bw/week), established by JECFA in 2011.

The estimated high-level weekly exposures to Al (*P*_50_, *P*_95,_ *P*_97.5_, and *P*_99_) are shown in Figure 2. The *P*_95_ is 2.0 mg/kg bw/week for young children, 1.0 mg/kg bw/week for children, 0.8 mg/kg bw/week for youths, and 0.6 mg/kg bw/week for adults. The percentages of *P*_95_ to PTWI (2.0 mg/kg bw/week) are 101% for young children, 49% for children, 40% for youths, and 31% for adults. As shown in Figure 2, high-level Al exposure (>*P*_95_) that exceeded the PTWI was observed only in small children. To limit Al intake in young children, the population with the highest-level Al exposure, the government should regulate the use of food additives, such as leavening agents (including baking powder) in the manufacture of confectionaries.

While it is likely that cooking utensils and food packaging contribute to Al intake, intake through kitchenware materials is considered to be less important than intake from food (Fekete et al. 2013). However, further studies investigating Al intake from cooking utensils and food packaging are necessary.

## Conclusion

The Al content of various food categories and the dietary estimated Al intake according to food category for young children, children, youths, and adults in Japan was determined using the market basket method, and Al analysis was conducted using ICP-AES. The Al content of samples of processed foods ranged from 0.40 to 21.7 mg/kg. For processed foods for all age groups, the Al content was highest in category VI samples, sugar and confections/savories, followed by category II samples, cereals. The daily dietary Al intake from processed foods is much larger than that from unprocessed foods. The average weekly dietary Al exposure from processed foods in young children, children, youths, and adults is estimated to be 0.86, 0.45, 0.35 and 0.30 mg/kg bw/week, respectively. High-level Al exposure (>*P*_95_) that exceeded the PTWI was observed only in young children.

## Supporting Information

**Table S1.** The levels of Al in the high Al content food category I and the daily dietary Al intake for all age groups. **Table S2.** The levels of Al in the high Al content food category II and the daily dietary Al intake for all age groups. **Table S3.** The levels of Al in the high Al content food category III and the daily dietary Al intake for all age groups.
