# Supplementary material for: Estimation of daily aluminum intake in Japan based on food consumption inspection results: impact of food additives
Source: Food Sci Nutr. 2014 Apr 20;2(4):389–97. doi: 10.1002/fsn3.114 (PMC4221837; doi:10.1002/fsn3.114)
Supplement: Supplementary file 1 [file fsn30002-0389-SD1.docx]

Table SuppInfo_1 The levels of Al in the high Al content food category І and the daily dietary Al intake for all age groups.

Table SuppInfo_2 The levels of Al in the high Al content food category Ⅱ and the daily dietary Al intake for all age groups.


Table SuppInfo_3 The levels of Al in the high Al content food category Ⅵ and the daily dietary Al intake for all age groups.
